# Antinuclear antibody and rheumatoid factor positivity in temporomandibular disorders

**DOI:** 10.1186/s13005-018-0183-3

**Published:** 2018-11-22

**Authors:** Ji Rak Kim, Jung Hwan Jo, Jin Woo Chung, Ji Woon Park

**Affiliations:** 1 Department of Dentistry and Oral Medicine, School of Medicine, Catholic University of Daegu, 33, Duryugongwon-ro 17-gil, Nam-gu, Daegu, 42472 Republic of Korea; 2 Department of Oral Medicine, Seoul National University Dental Hospital, 101, Daehak-ro, Jongno-gu, Seoul, 03080 Republic of Korea; 3 Department of Oral Medicine and Oral Diagnosis, School of Dentistry and Dental Research Institute, Seoul National University, 101, Daehak-ro, Jongno-gu, Seoul, 03080 Republic of Korea; 4 Department of Oral Medicine and Oral Diagnosis, School of Dentistry and Dental Research Institute, Seoul National University, 101, Daehak-ro, Jongno-gu, Seoul, 03080 Republic of Korea

**Keywords:** Temporomandibular joint disorders, Antinuclear antibodies, Rheumatoid factor, Autoimmunity, Hematologic tests

## Abstract

**Background:**

To investigate the differences in clinical characteristics and long-term treatment outcomes according to antinuclear antibody(ANA) and rheumatoid factor(RF) positivity and the correlation between pain-related and hematological indices in temporomandibular disorders(TMD) patients.

**Methods:**

Clinical examinations were done following the Research Diagnostic Criteria for TMD in 257 patients. Comprehensive screening along with psychological and hematological evaluations (ANA, RF, complete blood cell count, C-reactive protein[CRP] and erythrocyte sedimentation rate[ESR]) were conducted. Clinical characteristics and treatment outcomes were statistically compared between ANA/RF positive and negative groups.

**Results:**

Thirty-nine patients showed ANA/RF positivity. Male patients had smaller comfortable mouth opening(CMO)(*p* = 0.033) and maximum mouth opening(MMO)(*p* = 0.016) ranges with more painful neck muscles on palpation when RF/ANA positive. Pain duration, intensity, disability days and psychological distress levels were also higher in RF/ANA positive male patients. Significant correlation was shown in ESR with pain duration(*p* < 0.05) and numeric rating scale(NRS) before treatment(*p* < 0.05), CRP with NRS before treatment(*p* < 0.01), and red blood cell (RBC) with pain intensity(p < 0.05), NRS before treatment(p < 0.01), CMO(p < 0.01), pain on palpation of cervical muscles(p < 0.01), CMO(p < 0.05), and MMO(p < 0.05) 6 months after treatment.

**Conclusions:**

These results may point towards a nonspecific autoimmune disposition in a subgroup of TMD patients. RF and ANA could be considered as a screening test for the detection of autoimmune phenomena in TMD.

## Background

Temporomandibular disorders (TMD) is one of the most common musculoskeletal pain syndromes in the orofacial region mainly known as a clinical dysfunction of the temporomandibular joint (TMJ) and surrounding musculature that is accompanied by pain and movement limitation [1]. Most TMD patients respond well to conventional treatment including physical therapy and medication. Also intraoral appliances, surgical and orthodontic treatment can have a positive effect on patients with TMD including those with juvenile idiopathic arthritis (JIA) [2–4]. However a certain portion develop pain of a continuous and recurrent nature in addition to their alterations or limitations of mandibular movement [5]. Such patients show a higher prevalence of comorbidities such as headache, neuropathy, fatigue, and psychological problems. These symptoms can also be identified in pain disorders including complex regional pain syndrome, functional gastrointestinal disorders, fibromyalgia, and chronic fatigue syndrome suggesting the presence of a common pathophysiology. Recent studies speculate that chronic inflammation due to immune dysregulation involving autoimmunity could be the underlying cause of such chronic pain syndromes [6, 7]. Immune dysfunctions have also been associated with TMD of a longer duration and higher pain level [8].

Antinuclear antibody (ANA) and rheumatoid factor (RF) are laboratory tests applied to identify potential autoimmunity along with basic studies including complete blood cell count, acute phase reactants, and comprehensive metabolic panel [9]. ANA positivity has been associated with non-specific symptoms such as headache and general fatigue [10] and elevated levels have been reported in chronic fatigue syndrome [11] while higher RF incidence was shown in rheumatoid arthritis patients with persistent fatigue [12].

Currently the diagnosis of TMD is limited to radiographic and clinical examinations and there is a general lack of indices that may be applied to predict a patient’s prognosis [13]. There is a strong need to develop more reasonable and predictive markers to be applied in the diagnostic process. As in other pain disorders we hypothesized that the presence of ANA and RF would be related to the symptom severity of TMD. Therefore, the aim of this study was to investigate the presence of a possible autoimmune phenomena in TMD pain patients to eventually evaluate the potential role of ANA and RF as a diagnostic test for TMD and related disorders such as JIA by analyzing the differences in clinical characteristics and long-term treatment outcomes according to their positivity.

## Methods

### Subjects

The data of a total of 257 consecutive patients (51 men and 206 women, age range: 20–49 years, mean age: 29.54 ± 7.52 years) who visited the Orofacial Pain Clinic of Seoul National University Dental Hospital complaining of TMD symptoms from May, 2013 to July, 2015 were analyzed. Patients were diagnosed as TMD according to the Research Diagnostic Criteria for Temporomandibular Disorders (RDC/TMD) [14]. The clinical examination was performed by a single specialist on TMD and orofacial pain (J.W.P.) with more than 10 years of clinical experience.

Those with a history of other pain disorders within 6 months prior to the study, history of psychiatric or immune diseases, medication intake within 4 months prior to the study that could affect the results, history of recent trauma and orthognathic surgery, clinical history of other joint involvement, presence of inflammation or infection in other body parts were excluded from the study. Assessment was based on comprehensive screening and blood tests done before the RDC/TMD examinations. Patients with ANA and/or RF (ANA/RF) positivity were first referred to the department of rheumatology of Seoul National University Hospital and those who were diagnosed with a rheumatologic disease were also excluded. The included patients were divided into 2 groups according to the presence or absence of ANA/RF.

### Clinical assessment of TMD pain

All patients completed the RDC/TMD axis II questionnaire and a comprehensive interview concerning demographic and medical features including age, gender, pain-related characteristics (quality, duration, and intensity), general conditions (cardiovascular, musculoskeletal, psychological, and medication usage), and comorbidities of TMD (headache, sleep disturbance, neck and shoulder pain, lower back pain, arm and leg pain, and gastrointestinal disorder).

Clinical examination based on RDC/TMD included clinical parameters such as comfortable mouth opening (CMO), maximum mouth opening (MMO), pain on palpation of masticatory and cervical muscles and TMJ capsule, pain on mouth opening and eccentric mandibular movements (protrusion and laterotrusion). Overall TMD pain intensity was scored by the patient on a 0–10 numeric rating scale (NRS).

Pain-related disability level and the psychological status of patients were assessed by RDC/TMD axis II [15] and Symptom Checklist-90-Revision (SCL-90-R) [16].

### Quantification of autoantibodies and inflammatory markers

Plasma samples of all patients were obtained from the antecubital vein and stored in Lavender tubes coated with ethylenediaminetetraacetic acid (BD Vacutainer SST, Becton, Dickinson and company, Franklin Lakes, NJ, USA). The plasma was immediately centrifuged (3000 rpm) for 10 min at 4 °C, and analyzed. ANA was measured by indirect immunofluorescent methods using HEp2 cells (PhD IX, Bio-Rad Laboratories, Hercules, CA, USA). ANA titers of 1:40 or higher were considered as positive. RF was determined through enzyme-linked immunosorbent assay by detecting the presence of autoantibodies that react to the Fc portion of polyclonal IgM (Roche/Hitachi modular P800, Roche Diagnostics, GmbH, Mannheim, Germany).

Inflammatory markers including C-reactive protein (CRP) and erythrocyte sedimentation rate (ESR) were analyzed. Plasma concentrations of CRP were analyzed by means of a highly sensitive immunoturbidimetric assay autoanalyzer (Hitachi 7180, Hitachi High-Technologies Corp., Tokyo, Japan). Comprehensive laboratory assessments included complete blood cell counts with white blood cell differential, red cell indices, and blood chemistry. The person conducting serological analysis was blind to patients’ identity.

### Evaluation of treatment outcome

Following the initial examination all patients were educated about their TMD problems and instructions concerning the control of contributing factors (excessive muscle tension, bad posture, and parafunctional habits) of TMD were given. Demonstrations for self-exercise [17] and physical therapy were shown and the necessity of their regular implementation was emphasized. In addition, all patients had conservative management including occlusal stabilization splint and non-steroidal anti-inflammatory drugs. Patients were evaluated 6 months after the first treatment by the same examiner. Clinical parameters including CMO, MMO, pain on palpation of masticatory muscles and the TMJ capsule, and pain intensity on a 0–10 NRS were re-evaluated at this point.

### Statistical analysis

The Kolmogorov-Smirnov and Shapiro-Wilk test was applied to check for normality of the data. Non-parametric tests were applied when data were not normally distributed. Differences according to ANA/RF presence were analyzed by Mann-Whitney and Chi-square test. Correlations of each dimension of TMD clinical parameters, psychological profile and laboratory findings were analyzed by Spearman’s correlation coefficient. Results were considered statistically significant at a probability level of *p* < .05. All statistical analyses were performed with the SPSS 19.0 software program (IBM SPSS Inc., Chicago, IL, USA).

## Results

### Clinical characteristics and prognosis of TMD pain according to ANA/RF positivity

Two hundred fifty-seven patients completed assessment procedures at baseline and 6 months follow-up. Thirty-nine patients (15% of the total subjects) showed ANA/RF positivity.

The CMO(*p* = 0.033) and MMO(*p* = 0.016) ranges of ANA/RF positive male TMD patients were significantly smaller compared to ANA/RF negative male TMD patients before treatment initiation. ANA/RF positive male TMD patients had significantly more neck muscles that showed tenderness on palpation compared to ANA/RF negative male TMD patients. ANA/RF positive male TMD patients also had longer pain duration, higher pain intensity, and more disability days due to pain. The NRS before treatment was higher and a larger portion of patients reported a NRS higher than 5 compared to ANA/RF negative male TMD patients. ANA/RF positive male TMD patients more frequently reported pain during mouth opening and eccentric movement of the jaw. More masticatory muscles and TMJ capsule areas showed a positive response to palpation in ANA/RF positive TMD male patients although the differences were not statistically significant. The same tendencies of a higher pain level and resulting disability based on subjective and objective indices were identified in women TMD patients and the patient group as a whole but none of the differences was statistically significant according to ANA/RF positivity (Table 1).

**Table 1:** Temporomandibular disorders pain characteristics of the study population

	Total patients (*n* = 257)			Male patients (*n* = 51)			Female patients (*n* = 206)		
	ANA/RF (+) (*n* = 39)	ANA/RF (−) (*n* = 218)	*P*-value	ANA/RF (+) (*n* = 4)	ANA/RF (−) (*n* = 47)	P-value	ANA/RF (+) (*n* = 35)	ANA/RF (−) (*n* = 171)	P-value
Pain duration (months)^a^	12 (1.5–36)	3 (0.8–24)	0.117	27 (3.8–48)	3 (0.8–27)	0.233	12 (1–33)	3.5 (0.8–24)	0.215
Pain intensity^a^	45.0 (23.3–67.5)	46.7 (30–60)	0.822	48.3 (22.5–69.2)	47 (23.3–60)	0.699	45 (23.3–67.5)	46.7 (30–60)	0.620
Disability days^a^	4.5 (0–100)	10 (0–40)	0.454	100 (12.5–165)	10 (0–90)	0.355	4 (0–47.5)	10 (0–32.5)	0.260
NRS before^a^	6 (3–7)	5 (3–7)	0.384	6 (3.5–7.8)	5 (2.3–6)	0.244	6 (3–7)	5 (3–7)	0.723
NRS > 5^b^	26/39 (66.7%)	117/205 (57.1%)	0.292	3/4 (75%)	23/43 (53.5%)	0.617	23/35 (65.7%)	94/162 (58.0%)	0.452
Pain on opening^b^	20/39 (51.3%)	114/213 (53.5%)	0.862	2/4 (50%)	20/46 (43.5%)	1.000	18/35 (51.4%)	94/167 (56.3%)	0.709
Pain on eccentric movement^b^	20/39 (51.3%)	98/214 (45.8%)	0.602	2/4 (50%)	21/46 (45.7%)	1.000	18/35 (51.4%)	77/168 (45.8%)	0.580
Comorbidity^b^	19/39 (48.7%)	111/214 (51.9%)	0.731	1/4 (25%)	19/46 (41.3%)	0.641	18/35 (51.4%)	92/168 (54.8%)	0.852
CMO^a^	35 (30–46)	41 (32–47.3)	0.092	38.5 (28.5–41.8)	47 (42–54)	0.033*	33 (30–46)	39 (30–46)	0.479
MMO^a^	44 (33–49)	46 (40–50.3)	0.111	42 (31.5–48.8)	51 (47–55)	0.016*	44 (33–49)	45 (38–49)	0.783
Capsule palpation^a^	0 (0–1)	0 (0–2)	0.525	0.5 (0–4.8)	0 (0–1)	0.380	0 (0–1)	0 (0–2)	0.261
Muscle palpation -masticatory^a^	2 (0–7)	3 (0–6)	0.689	7.5 (1.5–9.8)	1 (0–4)	0.071	2 (0–6)	3 (1–6)	0.155
Muscle palpation -neck^a^	0 (0–4)	0 (0–2)	0.279	3 (0.5–7)	0 (0–1)	0.022*	0 (0–4)	0 (0–2)	0.861

ANA, antinuclear antibody; RF, rheumatoid factor; NRS, numeric rating scale; NRS before, NRS before treatment initiation; CMO, comfortable mouth opening; MMO, maximum mouth opening; Palpation, number of muscle that showed a positive response on palpation; Comorbidity, presence of at least one comorbidity

Pain intensity scores were calculated based on answers to the RDC/TMD Axis II questionnaire (mean score of question #7, 8, 9,10)

^a^Differences between group means were tested with Mann-Whitney test: Median (lower quartile - upper quartile)

^b^Differences between group means were tested with Chi-square test: number of subjects with a positive response/total number of subjects (percentage of subjects with a positive response)

*Significant difference: *p* < 0.05

Psychological profiles were various, and there were no statistically significant differences according to ANA/RF positivity in all patients (Table 2). However, the somatization, depression, and anxiety level was higher in ANA/RF positive TMD patients. After 6 months of conservative treatment the CMO of ANA/RF positive TMD patients was smaller (*p* = 0.024) and more ANA/RF positive female TMD patients reported pain during mouth opening (*p* = 0.022) compared to ANA/RF negative TMD patients. The significant differences in mouth opening range and number of positive neck muscles to palpation observed before treatment in male ANA/RF positive TMD patients had resolved to an insignificant level. However, mouth-opening ranges were still smaller and more patients reported pain on palpation of the capsule, neck and masticatory muscles compared to ANA/RF negative male TMD patients (Table 3).

**Table 2:** Psychological characteristics of the study population

	Total patients (*n* = 257)			Male patients (*n* = 51)			Female patients (*n* = 206)		
	ANA/RF (+) (*n* = 39)	ANA/RF (−) (*n* = 218)	*P*-value	ANA/RF (+) (*n* = 4)	ANA/RF (−) (*n* = 47)	*P*-value	ANA/RF (+) (*n* = 35)	ANA/RF (−) (*n* = 171)	*P*-value
RDC SOM	0.54 (0.08–1.20)	0.58 (0.17–1.25)	0.938	0.79 (0.27–1.56)	0.33 (0–0.92)	0.258	0.54 (0.08–1.17)	0.67 (0.25–1.29)	0.424
RDC PSOM	0.36 (0–0.91)	0.43 (0–1.07)	0.732	0.58 (0–18-1.61)	0.17 (0–0.70)	0.214	0.36 (0–0.89)	0.57 (0.14–1.14)	0.297
RDC DEP	0.60 (0.24–1.11)	0.55 (0.1–1.2)	0.606	0.50 (0.06–0.98)	0.20 (0–0.85)	0.798	0.60 (0.28–1.18)	0.64 (0.14–1.3)	0.822
SOM	43 (40–52)	43 (40–50)	0.546	48 (44–62)	43 (40–50)	0.142	43 (40–52)	43 (39–50)	0.801
O-C	42 (38–53)	42 (36–49)	0.381	46 (38–52)	41 (35–49)	0.529	42 (38–54)	42 (36–49)	0.473
I-S	41 (36–55)	41 (36–48)	0.762	47 (35–58)	40 (36–49)	0.736	41 (36–52)	41 (36–48)	0.840
DEP	41 (35–52)	40 (36–47)	0.879	41 (35–58)	39 (36–47)	0.966	41 (34–52)	41 (35–47)	0.992
ANX	43 (37–50)	40 (38–48)	0.322	51 (39–61)	40 (39–50)	0.369	43 (37–50)	41 (38–47)	0.395
HOS	43 (40–48)	43 (40–48)	0.651	47 (40–58)	42 (40–49)	0.595	43 (40–48)	43 (40–48)	0.737
PHOB	42 (40–48)	42 (40–48)	0.749	44 (41–62)	43 (43–45)	0.822	42 (40–48)	42 (40–48)	0.427
PAR	40 (38–42)	40 (38–42)	0.736	47 (39–56)	40 (38–42)	0.324	40 (38–42)	40 (38–43)	0.995
PSY	40 (38–52)	41 (38–46)	0.960	47 (39–58)	40 (40–45)	0.555	39 (38–47)	41 (38–46)	0.799

*ANA* antinuclear antibody, *RF* rheumatoid factor, *RDC SOM* somatization score of RDC/TMD axis II, *RDC PSOM* somatization score of RDC/TMD axis II without pain items, *RDC DEP* depression score of RDC/TMD axis II, *O-C* obsessive compulsive, *I-S* interpersonal sensitivity, *DEP* depression, *ANX* anxiety, *HOS* hostility, *PHOB* phobic anxiety, *PAR* paranoid ideation, *PSY* psychoticism

RDC SOM, PSOM, and DEP scores were calculated based on answers to the RDC/TMD Axis II questionnaire

Items not designated with RDC were based on the Symptom-Checklist-90-Revised

Differences between group means were tested with Mann-Whitney test: Median (lower quartile - upper quartile)

**Table 3:** Temporomandibular disorders pain characteristics of the study population after 6 months’ treatment

	Total patients (n = 257)			Male patients (n = 51)			Female patients (n = 206)		
	ANA/RF (+) (*n* = 39)	ANA/RF (−) (*n* = 218)	*P*-value	ANA/RF (+) (*n* = 4)	ANA/RF (−) (*n* = 47)	*P*-value	ANA/RF (+) (*n* = 35)	ANA/RF (−) (*n* = 171)	*P*-value
CMO^a^	42 (36–48)	45 (40–50)	0.024^*^	43 (37–53)	50 (45–53)	0.347	41 (35–48)	44 (40–48)	0.088
MMO^a^	44 (38–49)	46 (42–51)	0.069	47 (38–54)	52 (47–54)	0.246	44 (38–49)	45 (40–49)	0.325
Pain on opening^b^	17/39 (43.6%)	54/205 (26.3%)	0.035	1/4 (25.0%)	13/45 (28.9%)	1.000	16/34 (47.1%)	41/157 (26.1%)	0.022*
Capsule palpation^b^	9/39 (23.1%)	53/205 (25.9%)	0.842	2/4 (50.0%)	9/45 (20.0%)	0.214	6/33 (18.2%)	42/157 (26.8%)	0.381
Muscle palpation -masticatory^b^	12/39 (30.8%)	72/205 (35.1%)	0.714	3/4 (75.0%)	13/45 (28.9%)	0.096	8/33 (24.2%)	58/157 (36.9%)	0.227
NRS improvement^b^	23/33 (69.7%)	106/166 (63.9%)	0.557	4/4 (100%)	24/36 (66.7%)	0.297	19/28 (67.9%)	82/127 (64.6%)	0.829

*ANA* antinuclear antibody, *RF* rheumatoid factor, *CMO* comfortable mouth opening, *MMO* maximum mouth opening, *Palpation* a positive response on palpation, *NRS improvement* improvement of numeric rating scale score compared to the score before treatment initiation

^a^Differences between group means were tested with Mann-Whitney test: Median (lower quartile - upper quartile)

^b^Differences between group means were tested with Chi-square test

### Hematological characteristics according to ANA/RF positivity

ANA/RF positive male TMD patients (*p* = 0.001) and TMD patients as a whole group (*p* = 0.037) had a significantly higher ESR level compared to their ANA/RF negative counterparts. This difference was not significant in female TMD patients. ANA/RF positive male TMD patients had lower red blood cell counts, hemoglobin levels, and higher CRP levels compared to ANA/RF negative patients although the difference was not statistically significant (Table 4).

**Table 4:** Hematological characteristics of the study population

	Total patients (*n* = 257)			Male patients (*n* = 51)			Female patients (n = 206)		
	ANA/RF (+) (*n* = 39)	ANA/RF (−) (*n* = 218)	*P*-value	ANA/RF (+) (*n* = 4)	ANA/RF (−) (*n* = 47)	*P*-value	ANA/RF (+) (*n* = 35)	ANA/RF (−) (*n* = 171)	*P*-value
White Blood Cell (10^3^/ul)	5.5 (4.9–7.3)	5.9 (5.0–7.0)	0.697	6.1 (4.6–7.8)	5.6 (4.6–6.9)	0.639	5.5 (4.9–6.9)	6.1 (5.0–7.0)	0.464
Red Blood Cell (10^6^/ul)	4.4 (4.2–4.7)	4.5 (4.2–4.9)	0.086	4.7 (4.1–5.2)	5.1 (4.9–5.3)	0.125	4.4 (4.2–4.6)	4.4 (4.2–4.6)	0.853
Hemoglobin (g/dl)	13.0 (12.7–14.2)	13.5 (12.8–14.5)	0.163	14.9 (13.3–15.9)	15.6 (15.0–16.1)	0.209	13.0 (12.7–14.0)	13.1 (12.6–13.8)	0.938
Platelet (10^3^/ul)	238 (198–300)	251 (217–278)	0.463	252 (217–272)	236 (218–265)	0.623	238 (195–300)	256 (216–285)	0.306
ESR (mm/hr)	6 (4–13)	5 (2–10)	0.037*	7 (6–8.8)	2 (2–4)	0.001*	6 (3–14)	7 (3–12)	0.447
CRP (mg/dl)	0.05 (0.03–0.11)	0.05 (0.04–0.10)	0.787	0.14 (0.07–0.20)	0.06 (0.04–0.10)	0.068	0.05 (0.03–0.08)	0.05 (0.04–0.08)	0.888

*ANA* antinuclear antibody, *RF* rheumatoid factor, *ESR* erythrocyte sedimentation rate, *CRP* C-reactive protein

Differences between group means were tested with Mann-Whitney test: Median (lower quartile - upper quartile)

*Significant difference: *p* < 0.05

### Relationship between hematological and TMD pain-related indices

Significant correlation was shown between RBC with pain intensity (γ = − 0.297, *p* < 0.05), NRS before treatment (γ = − 0.402, *p* < 0.01), CMO (γ = 0.365, *p* < 0.01), number of positive muscles on palpation of cervical muscles (γ = − 0.393, *p* < 0.01), CMO at 6 months after treatment (γ = 0.323 p < 0.05), and MMO at 6 months after treatment (γ = 0.331, p < 0.05). Hemoglobin concentration showed significant correlation with MMO at 6 months after treatment (γ = 0.303, *p* < 0.05). ESR showed significant correlation with pain duration (γ = − 0.279, p < 0.05) and NRS before treatment (γ = 0.297, *p* < 0.05) and CRP with NRS before treatment (γ = 0.417, *p* < 0.01) (Table 5). There were no significant correlations between laboratory findings and psychological indices (data not shown).

**Table 5:** Correlations among hematological and pain-related indices in male temporomandibular disorders patients

	Pain duration	Pain intensity	Disability days	NRS before	CMO	MMO	Capsule palpation	Muscle palpation -masticatory	Muscle palpation-neck	CMO6	MMO6
WBC (10^3^/ul)	−0.240	0.069	0.106	0.110	−0.071	−0.022	0.259	−0.182	− 0.011	−0.031	− 0.067
RBC (10^6^/ul)	0.073	−0.297^*^	− 0.260	−0.402^**^	0.365^**^	0.267	−0.143	− 0.262	−0.393^**^	0.323^*^	0.331^*^
Hemoglobin(g/dl)	0.087	−0.130	0.040	−0.228	0.249	0.248	−0.191	−0.208	− 0.140	0.264	0.303^*^
Platelet (10^3^/ul)	0.020	0.008	−0.009	0.081	−0.088	−0.016	− 0.110	−0.215	− 0185	0.081	0.033
ESR (mm/hr)	−0.279^*^	0.132	−0.039	0.297^*^	− 0.175	−0.155	0.082	−0.061	0.091	−0.130	− 0.163
CRP (mg/dl)	−0.207	0.226	0.040	0.417^**^	− 0.217	−0.097	0.006	0.020	0.202	−0.066	−0.075

*NRS before* numeric rating scale score before treatment initiation, *CMO* comfortable mouth opening, *MMO* maximum mouth opening, *Palpation* number of muscle that showed a positive response on palpation, *CMO6* CMO following 6 months of treatment, *MMO6* MMO following 6 months of treatment, *WBC* white blood cell, *RBC* red blood cell, *ESR* erythrocyte sedimentation rate, *CRP* C-reactive protein

Pain intensity scores were calculated based on answers to the RDC/TMD Axis II questionnaire (mean score of question #7, 8, 9*10)

^*^Significant difference: p < 0.05

^**^Significant difference: *p* < 0.01

## Discussion

The results of this study showed that male TMD patients with ANA/RF positivity were associated with higher pain levels and more clinical dysfunction followed by worse long-term treatment outcomes. This study provided evidence of a possible role of autoimmunity in the pathogenesis of TMD pain in a certain patient subgroup. RF positivity has been reported to be found in around 4% [18] while ANA positivity can be identified in around 5% of the general healthy population [19]. Considering this the ANA/RF positivity rate (15%) of this study may not be an alarming number but the fact that ANA/RF positivity may appear years before being diagnosed with a definitive autoimmune disease should be considered seriously [20]. It could be said that ANA/RF positive TMD male patients are vulnerable to higher pain levels and resultingly suffer from more functional problems of the TMJ. And not only markers of autoimmunity but also routine laboratory indices and markers of inflammation appear to be associated with pain-related indices. It is known that autoimmune markers can act as a predictor of clinical symptoms in several chronic pain syndromes. Studies show that pain in complex regional pain syndrome can result from an autoimmune process [21] and a high percentage of fibromyalgia patients display autoimmunity leading to immunological aberration [22]. However, the possibility of autoimmunity as an etiologic factor in TMD pathogenesis has rarely been sought out. One study exists that showed increased failure of TMJ surgery in patients with ANA/RF positivity [23].

The ANA/RF positive TMD patients also showed higher scores of anxiety and depression. An intimate association between autoimmunity and depression has been demonstrated [24] and reports show that autoimmunity may be a crucial risk factor of anxiety disorder [25]. TMD is also a chronic pain disorder well known to be accompanied by psychological problems such as depression and anxiety [26]. This suggests that certain clinical symptoms of TMD including pain and psychological distress may be a reflection of the underlying chronic inflammation which is caused by an autoimmune process.

ANA/RF positive male TMD patients of this study had lower red blood cell counts and hemoglobin levels showing an anemic tendency. Patients with autoimmune disorders have been reported to show altered hematologic conditions. Autoantibodies may bind to antigens expressed on RBC surfaces and initiate their destruction through the complement system. An example is immune hemolytic anemia which is a disorder characterized by decreased RBC or RBC-related indices due to autoimmune destruction [27]. Autoimmune thrombocytopenic purpura is a clinical condition with increased platelet-immunoglobulin and accelerated destruction of platelets resulting in bleeding disorders [28].

ESR is a non-specific measure of inflammation which is increased in inflammatory states. In our study, ANA/RF positive men showed significantly higher ESR levels. Increased ESR may represent a chronic inflammatory condition associated with elevated pro-inflammatory cytokine levels. Such inflammatory conditions can directly result in various pain-related TMD symptoms [29] since inflammation has been directly associated with various pain symptoms including sleep disorders [30] and heightened sensitivity [31]. The fact that ESR levels in this study were closely associated with NRS scores and pain duration may also support the possibility of a causal relationship between pain and inflammation. CRP, another marker of inflammation is also significantly related with NRS scores. Patients with higher ESR and CRP levels may be closely related to TMD pain of a higher level and longer duration. Although indirect these results suggest the possibility of chronic inflammation as a contributing factor in the pathogenesis of TMD pain and since such markers were elevated in the ANA/RF positive TMD group we may speculate that autoimmunity could play a part in such inflammation.

Correlations among hematological and TMD pain-related indices were revealed in the male TMD patients. Among them, RBC was most closely associated with TMD pain-related indices. Decreased RBC levels were related to increased pain levels and impaired mandibular function. This may reflect the relationship among autoimmunity, anemia, and pain since higher hemoglobin concentrations were also significantly related to larger CMO values after 6 months’ of treatment in ANA/RF positive male TMD patients.

Most autoimmune disorders are significantly more common in women [32, 33]. Female predominance is assumed to be due to hormonal differences with estrogens and androgens playing a potential role in the autoimmune process [34]. However, the exact pathogenesis is yet to be fully elucidated. The reason why most significant findings according to ANA/RF positivity were evident only in male patients is not easy to explain considering the complex and multifactorial etiology of autoimmune phenomena. The frequency of ANA positivity is known to differ according to gender with females showing a higher positivity rate [35]. A higher rate of RF positivity has been reported in males compared to females in rheumatoid arthritis patients [36]. Such studies support the fact that ANA/RF positivity is influenced by gender and this must be considered in selecting such indices for diagnostic purposes and interpreting test results. The pathophysiology of TMD may be affected by various factors including gender and it is generally accepted that women are more easily afflicted than men [37]. Women have more confounding factors that may influence pain levels such as hormonal fluctuations following the menstrual cycle and a higher prevalence of psychological problems compared to men. This may have diluted the influence of inflammation due to autoimmunity on TMD pain resulting in insignificant differences according to ANA/RF positivity. Prevalence of ANA/RF positivity is known to increase with age [33, 35]. To minimize such effects, we limited the investigation to patients with an age range of 20–49 years.

Many studies discuss the pathophysiology of TMD but the complexity of the underlying mechanism hinders complete understanding and a consensus concerning this issue is yet to be reached. So the diagnostic process of TMD is usually based on empirical evidence and is mostly constituted of interviews, physical examinations, and imaging [13]. However, such approaches fall short in differentiating TMD patients according to pathophysiology and cannot be used to predict treatment outcomes and disease prognosis. So the development of a reliable diagnostic tool that will accurately reflect a patient’s pain level and assist in patient grouping is highly called upon. This is the first longitudinal study showing the predictive values of laboratory tests in differentiating TMD patients with different pain and jaw dysfunction levels. Based on the results of this study, we can see a putative role of laboratory examinations to point out specific groups that may share an autoimmune pathophysiology and show higher pain levels and disability in the diagnostic process of TMD.

At the baseline, most clinical indices of TMD patients with ANA/RF positivity were significantly worse compared to ANA/RF negative subjects. However, after 6 months of conservative therapy TMD pain levels also improved in ANA/RF positive patients and the difference in symptom severity was no longer significant between the two groups. This may reflect the effectiveness of conservative treatment in controlling TMD symptoms. TMD patients with autoimmune tendency may have a more painful functional disorder but they also responded well to conservative therapy. So the application of conservative treatment could be advised regardless of ANA/RF positivity. But the dysfunction level of ANA/RF positive patients were still higher in spite of long-term treatment. This may suggest the possibility of using ANA and RF in predicting patient response to conservative treatment at the initial evaluation, allowing for more active measures to be taken at an earlier stage for certain TMD patients that may not respond well to conservative approaches. However, the results of this study have certain limitations.

First the small sample size of ANA/RF positive patients could have lowered statistical power resulting in insignificant results. Further studies of a larger scale and longer duration must be conducted to evaluate the reliability of ANA and RF as diagnostic indices in TMD. Secondly comorbidities are known to affect TMD pain levels [38, 39] but exclusion of subjects with other pain disorders was solely based on interviews lacking an independent examination based on specific diagnostic criteria. Third, although the results are based on reliable diagnostic processes and the study was conducted in a longitudinal fashion the results cannot be said to directly show the role of autoimmunity in TMD pain generation. Future studies should be carried out by analyzing numerous markers of autoimmunity and inflammation in more specific TMD subgroups to support a causal relationship between autoimmunity and TMD pain. Applying the sera of TMD patients who show autoimmunity to pain related structures such as the dorsal horn and checking for responsiveness should be a good approach [40]. The assessment of ANA pattern and its correlation with clinical characteristics could be another approach.

## Conclusions

This study evaluated differences in clinical symptoms of TMD according to ANA/RF positivity and the results suggest a possible role of autoimmunity and inflammation in the pathophysiology of TMD pain and resulting dysfunction. It might not be possible to apply these results to all patients but a certain group of TMD patients may show an autoimmune disposition that is associated with pain of a higher level and more dysfunction followed by poor treatment outcomes. Such results may be considered in the diagnosis and prognosis prediction process of chronic TMD pain and hematologic indices such ANA and RF should be further evaluated.

## Abbreviations

ANA: Antinuclear antibody
CMO: Comfortable mouth opening
CRP: C-reactive protein
ESR: Erythrocyte sedimentation rate
MMO: Maximum mouth opening
NRS: Numeric rating scale
RBC: Red blood cell
RDC/TMD: Research Diagnostic Criteria for Temporomandibular Disorders
RF: Rheumatoid factor
SCL-90-R: Symptom Checklist-90-Revision
TMD: Temporomandibular disorders
TMJ: Temporomandibular joint
